# A past medical history of heart failure is associated with less fluid therapy in septic patients

**DOI:** 10.5935/0103-507X.20190049

**Published:** 2019

**Authors:** Carlos Rodrigo Franco Palacios, Amanda M. Thompson, Federico Gorostiaga

**Affiliations:** 1 Hospital Medicine and Nephrology Department, Carris Health, Rice Memorial Hospital - Willmar, Minnesota, United States.; 2 Pulmonary and Critical Care Medicine Department, Jackson Memorial Hospital, University of Miami - Miami, Florida, United States.; 3 Pharmacy Department, Carris Health, Rice Memorial Hospital - Willmar, Minnesota, United States.; 4 Critical Care Department, Mount Sinai Medical Center - Miami Beach, Florida, United States.

**Keywords:** Sepsis, Heart failure, Fluid therapy

## Abstract

**Objective:**

To identify the underlying factors that affect fluid resuscitation in septic patients.

**Methods:**

The present study was a case-control study of 181 consecutive patients admitted to a Medical Intensive Care Unit between 2012 and 2016 with a diagnosis of sepsis. Demographic, clinical, radiological and laboratory data were analyzed.

**Results:**

One hundred-thirty patients (72%) received ≥ 30mL/kg of IV fluids on admission. On univariate analyses, a past history of coronary artery disease and heart failure was associated with less fluid therapy. On multivariate analyses, a history of heart failure (OR = 2.31; 95%CI 1.04 - 5.14) remained significantly associated with receiving less IV fluids. Left ventricular ejection fraction, systolic/diastolic function, left ventricular hypertrophy and pulmonary hypertension were not associated with IV fluids. The amount of IV fluids was not associated with differences in mortality. During the first 24 hours, patients with a past history of heart failure received 2,900mLof IV fluids [1,688 - 4,714mL] *versus* 3,977mL [2,500 - 6,200mL] received by those without a history of heart failure, p = 0.02.

**Conclusion:**

Septic patients with a past history of heart failure received 1L less IV fluids in the first 24 hours with no difference in mortality.

## INTRODUCTION

Administering 30mL/kg of crystalloids for hypotension or ≥ 4mmol/L lactate is recommended in the latest Surviving Sepsis Campaign guidelines for the treatment of sepsis and septic shock. Other measures include obtaining serum lactate and blood cultures prior to antibiotics and broad-spectrum antibiotics.^(1-3)^

The rationale for fluid resuscitation in sepsis is supported by the fact that the peak activity of the inflammatory response appears between 1 and 6 hours after an insult. In human models of endotoxemia, isotonic prehydration significantly decreases the concentration of proinflammatory cytokines and increases the concentration of anti-inflammatory cytokines.^(4)^

Since compliance with sepsis bundles is variable among providers, even in specialties that normally care for septic patients, and intravenous (IV) fluids - IVF - are an early component of sepsis treatment, the factors that affect their implementation warrant investigation.^(5)^

The objective of this study was to identify the underlying variables associated with fluid therapy in sepsis. We hypothesized that certain patient characteristics present at the time of admission would influence the amount of IVF resuscitation.

## METHODS

After obtaining approval from the Rice Memorial Hospital Institutional Review Board, a retrospective case-control study of 181 consecutive patients newly admitted to the intensive care unit (ICU) between 2012 and 2016 with a diagnosis of sepsis was carried out.

This study period was chosen because the hospital started using a common Electronic Medical Record software in 2012, allowing for easier access to data.

The ICU was staffed by either a dedicated hospitalist team or family medicine physicians.

Sepsis was defined as a documented or suspected infection with two of the following: temperature of less than 36ºC or more than 38ºC, a leukocyte count of < 4 × 109/L or more than 12 × 109/L, respiratory frequency of > 20 breaths per minute or mechanical ventilation and a heart rate of more than 90 beats per minute or a sequential (sepsis related) organ failure assessment (Sequential Organ Failure Assessment - SOFA) score ≥ 2. Septic shock was defined as the above plus plasma lactate of more than 2mmol/L (in the absence of hypovolemia) and requirement of a vasopressor infusion to maintain a mean arterial blood pressure (MAP) of 65mmHg after initial fluid resuscitation.^(6)^

The inclusion criteria were septic patients newly admitted to the ICU who were hypotensive upon first evaluation (systolic blood pressure - SBP < 100mmHg or MAP < 65mmHg) and or had ≥ 4mmol/L lactic acid since these patients qualified for IV fluid resuscitation according to guidelines.

The cases were patients who received less than 30mL/kg of IVF in the first 24 hours after diagnosis of sepsis. The controls were patients who received ≥ 30mL/kg of IVF in the first 24 hours after diagnosis of sepsis.

The exclusion criteria were pregnant patients, patients younger than 18 years, patients not admitted to the ICU, patients presenting to the Emergency Room with pulmonary edema (on radiological studies) or decompensated congestive heart failure (jugular vein distention, bilateral pulmonary crackles, lower extremity edema with or without elevated B-type natriuretic peptide levels), and patients with end stage renal disease requiring dialysis.

Demographic, clinical, radiological and laboratory data were collected. Echocardiographic data were based on two-dimensional echocardiogram results (if done) at baseline (prior to hospitalization).

New onset pulmonary edema (not present before admission) was diagnosed by readings of chest radiography or a computed tomography scan of the chest.

### Statistical analyses

Data are presented as the mean and standard deviation if normally distributed and the median [25% and 75% percentiles] or range if not normally distributed. For parametric data, differences in the mean were compared by Student's t-test. For highly skewed data, the Wilcoxon-Mann-Whitney test was used. The normality of the data was assessed by a frequency distribution histogram.

Differences in proportions were assessed by the chi square test or Fisher's exact test.

Multivariate logistic regression models were used to study associations and adjust for confounding factors. Underlying medical conditions with p-values ≤ 0.1 in univariate analyses were included. Survival curves were generated using the Kaplan-Meier method.

P-values lower ≤ 0.05 were considered statistically significant. All analyses were performed using JMP statistical software version 13 (SAS Campus Drive, Cary, NC).

## RESULTS

The baseline characteristics are described in table 1. One hundred-thirty patients (72%) received ≥ 30mL/kg of IVF on admission day.

**Table 1:** Baseline characteristics

Characteristics	≥ 30mL/kg IV fluids N = 130	< 30mL/kg IV fluids N = 51	p value
Age (years)	68 [59 - 78]	74 [66 - 80]	0.07
Male gender	64 (49.2)	23 (45.1)	0.62
Congestive heart failure	25 (19.2)	20 (39.2)	0.007
Ejection fraction	60 [55 - 65]	60 [58.7 - 65]	0.86
Left ventricular systolic dysfunction*	12 (14.3)	7 (18.4)	0.59
Left ventricular diastolic dysfunction†	41 (57.7)	15 (62.5)	0.81
Left ventricular hypertrophy‡	42 (50)	24 (64.8)	0.16
Pulmonary hypertension§	31 (37.3)	16 (45.7)	0.41
Diabetes mellitus	45 (34.6)	18 (35.3)	1
Hypertension	86 (66.1)	36 (70.6)	0.60
Chronic kidney disease	35 (26.9)	12 (23.5)	0.70
Coronary artery disease	28 (21.5)	19 (37.2)	0.038
Atrial fibrillation	25 (19.2)	14 (27.4)	0.23
Cancer	32 (24.6)	16 (31.4)	0.35
Pulmonary disease	54 (41.5)	20 (39.2)	0.86
Chronic liver disease	6 (4.62)	6 (11.7)	0.1
MAP upon ICU admission (mmHg)	71 [61 - 86]	76 [65 - 90]	0.052
Positive blood culture during hospitalization	45 (34.6)	24 (47)	0.08
Time to vasopressor use (hours)	5 [2 - 9]	6 [3 -16]	0.15
Admission creatinine (mg/dL)	1.32 [0.83 - 1.97]	1.34 [1.00 - 1.97]	0.25
Admission lactic acid (mg/dL)	2.0 [1.4 - 3.4]	1.5 [1.1 -2.6]	0.15
Admission SOFA score	8 [5 - 10]	7 [5 - 9]	0.29

IV - intravenous; MAP - mean arterial blood pressure; ICU - intensive care unit; SOFA - sequential organ failure assessment score.

* Left ventricular systolic dysfunction data were available for 84 patients in group 1 and 38 patients in group 2.

† Left ventricular diastolic dysfunction data were available for 71 patients in group 1 and 24 patients in group 2.

‡ Left ventricular hypertrophy data were available for 84 patients in group 1 and 37 patients in group 2.

§ Pulmonary hypertension data were available for 83 patients in group 1 and 35 patients in group 2. Results expressed as median [interquartile range] or N (%).

Pneumonia was the cause of sepsis in sixty-three patients (34.2%), urinary tract infections were the cause in 41 patients (22.3%), cellulitis and peritonitis were the cause in 8 patients (4.3%), cholecystitis was the cause in 6 patients (3.2%), *Clostridium difficile* colitis was the cause in 6 patients (3.2%), endocarditis was the cause in 5 patients (2.7%), and cholangitis was the cause in 4 patients (2.1%).

Twenty-one patients (11.4%) had nonspecified sepsis.

One hundred twenty-six patients (70.7%) received isotonic crystalloids (either lactate ringer or normal saline), 9 patients (5%) received hypotonic fluids (either D5W or half normal saline), and 43 patients (24.1%) received a mix of different isotonic crystalloids. Data were missing for 3 patients.

Other causes of sepsis included catheter infections, bowel ischemia, bowel perforation, diverticulitis, necrotizing fasciitis, neutropenic fever, osteomyelitis, pelvic inflammatory disease, perirectal abscess, septic arthritis, thrombophlebitis and toxic shock syndrome.

Patients who received at least 30mL/kg of IVF upon admission had a more positive fluid balance. No difference in survival or pulmonary edema (radiographic) was noted between groups (Table 2).

**Table 2:** Outcomes

Outcome	≥ 30mL/kg IV fluids N = 130	< 30mLkg IV fluids N = 51	p value
Amount of fluids given on admission day (L)	4.6 [3.51 - 6.73]	1.7 [1.22 - 2.38]	< 0.0001
Amount of fluids given on admission day (mL/kg)	59.8 [43.1 - 72.7]	20.7 [13.2 - 25.7]	< 0.0001
Hospitalization total fluid balance (L)	7.92 [4.2 - 12.3]	4.71 [2.65 - 9.22]	0.01
Pulmonary edema on chest radiography	38 (29.2)	20 (39.2)	0.21
Vasopressor use during ICU stay	78 (60)	20 (39.2)	0.01
Mechanical ventilation during ICU stay	33 (25.4)	12 (23.5)	1
AKI during hospitalization	82 (63)	35 (68.6)	0.36
Renal replacement therapy during hospitalization	3 (2.3)	1 (1.9)	1
ICU days	2 [1 - 4]	2 [1 - 3]	0.03
Hospital days	5 [3 - 11]	5 [3 - 7]	0.30
Alive at discharge	105 (80.7)	40 (78.4)	0.68
Alive at day 30	92 (76.7)	34 (69.4)	0.33
Alive at day 90	83 (71.5)	33 (67.3)	0.58

IV - intravenous; ICU - intensive care unit; AKI - acute kidney injury. Acute kidney injury is based on the AKIN definition. Stage 1: increased serum creatinine ≥ 0.3mg/dL or increased serum creatinine ≥ 150 a 200% (1.5 a 2×) or urine output < 0.5mL/kg/hour (> 6 hours). Stage 2: increased serum creatinine > 200 a 300% (> 2 a 3×) or urine output < 0.5mL/kg/hour (> 12 hours). Stage 3: increased serum creatinine > 300% (> 3×) or if baseline serum creatinine ≥ 4mg/dL increased serum creatinine ≥ 0.5mg/dL or urine output < 0.3mL/kg/hour (24 hours) or anuria (12 hours) or need for renal replacement therapy. Results expressed as median [interquartile range] or N (%).

A past medical history of congestive heart failure (CHF) and coronary artery disease (CAD) and a current need for vasopressor use were associated with the amount of IVF resuscitation according to univariate analyses.

Of the underlying medical conditions, after adjusting for confounding variables, a history of CHF remained significantly associated with IVF therapy (Table 3).

**Table 3:** Multivariate analyses of underlying medical conditions associated with less fluid resuscitation

Variable	Odds Ratio	95%CI	p value
Age (years)	1.007	0.98 - 1.03	0.51
Congestive heart failure	2.31	1.04 - 5.14	0.03
Coronary artery disease	1.39	0.60 - 3.22	0.43
Chronic liver disease	3.17	0.93 - 10.7	0.06

95%CI - 95% confidence interval.

### History of congestive heart failure and fluid therapy

**Figure 1:**
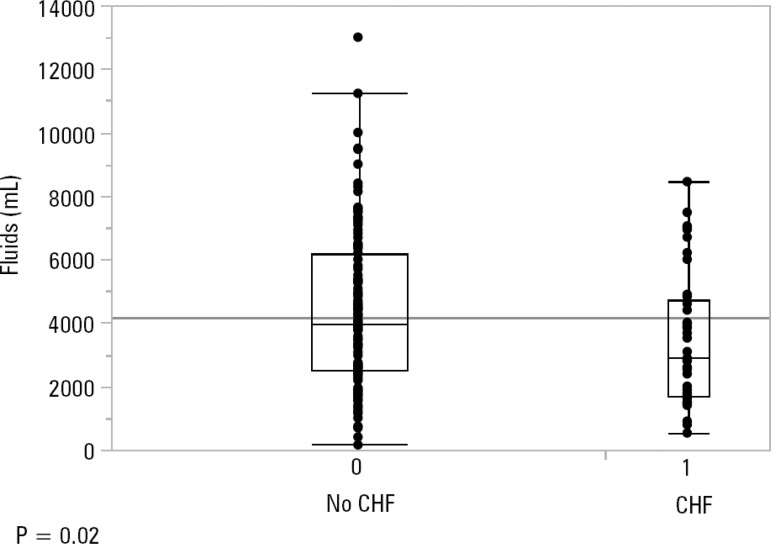
Amount of fluids given on admission. CHF - congestive heart failure.

Upon admission, patients with a history of CHF received 2,900 cc of IVF [1,688 - 4,714] *versus* 3,977mL [2500 - 6200] received by those without CHF, p = 0.02 (Figure 1).

The total fluid balance in patients with CHF was 5,320mL [2,430 - 10,357] *versus* 6,776mL [3,900 - 12,417] in those without CHF, p = 0.04.

Left ventricular ejection fraction, systolic/diastolic function, left ventricular hypertrophy and pulmonary hypertension were not associated with IVF (Table 1).

**Figure 2:**
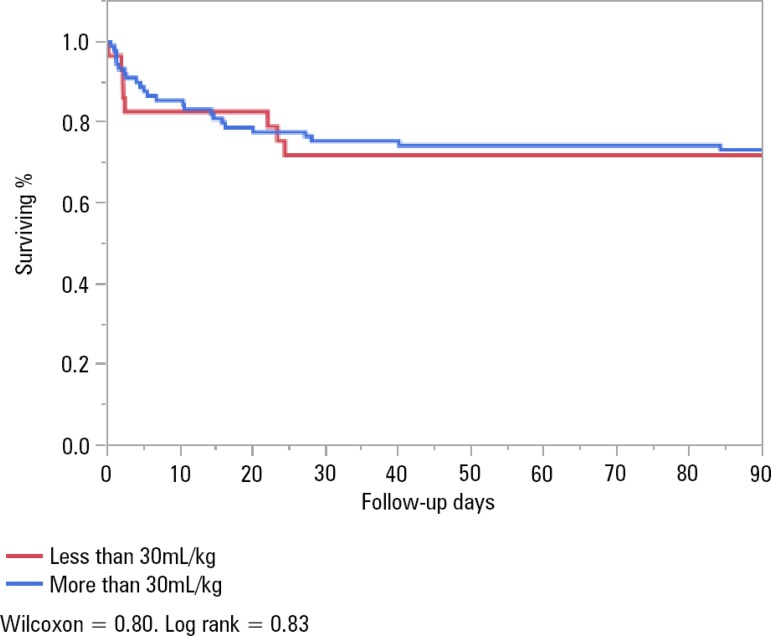
Survival analysis of patients with no history of congestive heart failure.

Survival in patients without CHF who received ≥ 30cc/kg of IVF *versus* those who did not was 78.6% *versus* 77.4%, p = 1, upon discharge; 75.8% *versus* 70%, p = 0.63, at 30 days; and 72.5% *versus* 70%, p = 0.81, at 90 days (Figure 2), respectively.

**Figure 3:**
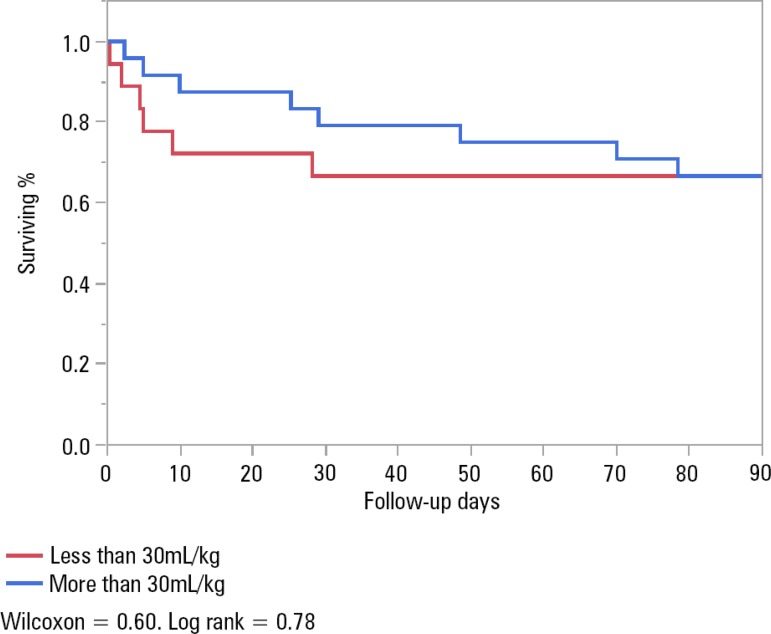
Survival analysis of patients with history of congestive heart failure.

Survival in patients with a history of CHF who received ≥ 30mL/kg of IVF *versus* those who did not was 92% *versus* 80%, p = 0.38, upon discharge; 79.1% *versus* 68.4%, p = 0.49, at 30 days; and 66.7% *versus* 63.2%, p = 1, at 90 days (Figure 3), respectively.

## DISCUSSION

The purpose of this study was not to test the benefits or harms of IV fluids in septic patients but to merely understand the underlying patient characteristics that affect fluid therapy in sepsis. To date, there is a paucity of data to explain why some patients receive less fluid resuscitation than others, with most studies only describing variations in treatment and not the reasons that led to the treatment decisions.

We found that in septic patients admitted to the ICU in a rural community hospital, a past medical history of CHF was independently associated with less fluid therapy.

Compliance with all components of a sepsis bundle is highly variable, even within the same hospital at different times of day. Nonetheless, antibiotics and IV fluids tend to be completed more often (in approximately one-third of patients).^(7-9)^

In the present study, the amount of fluid resuscitation measured was not restricted to the first 3 hours due to inherent challenges in how hourly input and output were charted. Instead, the amount of IVF given within the first 24 hours of sepsis diagnosis was used for analyses. However, there is evidence that completion of a treatment bundle before 18 hours is associated with 10% decreased hospital mortality.^(10)^

Prior studies have shown that treatment after bundle implementation is associated with improved hospital survival in patients with heart failure. We found no better hospital or short-term survival in these patients when more fluids were given.^(11,12)^

In our study, after adjusting for cofounding variables, the odds of receiving less fluid therapy were more than 2 times higher in patients with a past medical history of CHF. These patients received 1L less of IV fluids when sepsis was diagnosed. This difference could be explained by concerns regarding fluid overload and pulmonary edema in patients with a history of cardiac disease.

Septic patients are prone to extracellular fluid accumulation and pulmonary edema due to increased capillary permeability and increased intravascular hydrostatic pressure. Many of these patients develop fluid overload with the amount of fluid recommended in the current early goal-directed therapy (EGDT) guidelines. Bioimpedance vector analysis of patients admitted to the ICU for treatment of sepsis showed that > 90% developed volume overload, which is associated with an increased need for diuresis and thoracentesis, worsening renal function and increased mortality.^(13-15)^

In this study, a more positive fluid balance and weight gain was found in patients who received ≥ 30mL/kg of IVF but not more pulmonary edema or acute kidney injury.

Furthermore, recent randomized multicenter trials have challenged the usefulness of the EGDT bundle, with no difference in survival noted compared to standard care bundles.^(16-18)^

In this study, septic patients who received ≥ 30mL/kg of IVF also received more vasopressors. This difference may indicate a more aggressive treatment approach. Another possibility is that these patients were more acutely ill, even though their admission SOFA scores were similar and there was no difference in survival.

The limitations of this study include it being a retrospective, single-center study with a predominantly Caucasian population in a rural setting with a relatively small number of patients and a lack of a dedicated intensivist team. Most rural hospitals in the country face the same challenges.^(19)^

## CONCLUSION

Septic patients with a past history of congestive heart failure received 1L less of IV fluids on admission compared to those without a history of congestive heart failure. In spite of this difference, no difference in mortality was noted.
